# Transcriptomic response is more sensitive to water deficit in shoots than roots of *Vitis riparia* (Michx.)

**DOI:** 10.1186/s12870-019-1664-7

**Published:** 2019-02-13

**Authors:** Vedbar Singh Khadka, Kimberley Vaughn, Juan Xie, Padmapriya Swaminathan, Qin Ma, Grant R. Cramer, Anne Y. Fennell

**Affiliations:** 1 McFadden BioStress Laboratory, Agronomy, Horticulture, and Plant Science Department, South Dakota State University, Brookings, SD 57006 USA; 2 JABSOM Bioinformatics Core, Department of Complementary & Integrative Medicine, University of Hawaii, Honolulu, HI USA; 3 South Dakota State University, Brookings, SD 57006 USA; 4 Department of Biochemistry and Molecular Biology, University of Nevada, Reno, NV USA

**Keywords:** Drought, Grapevine, ABA, ABF2, ABF3, NAC, Circadian rhythm, Cytokinin, Signaling network, *Vitis riparia*

## Abstract

**Background:**

Drought is an important constraint on grapevine sustainability. *Vitis riparia,* widely used in rootstock and scion breeding, has been studied in isolated leaf drying response studies; however, it is essential to identify key root and shoot water deficit signaling traits in intact plants. This information will aid improved scion and rootstock selection and management practices in grapevine. RNAseq data were generated from *V. riparia* roots and shoots under water deficit and well-watered conditions to determine root signaling and shoot responses to water deficit.

**Results:**

Shoot elongation, photosynthetic rate, and stomatal conductance were significantly reduced in water deficit (WD) treated than in well-watered grapevines. RNAseq analysis indicated greater transcriptional differences in shoots than in roots under WD, with 6925 and 1395 genes differentially expressed, respectively (q-value < 0.05). There were 50 and 25 VitisNet pathways significantly enriched in WD relative to well-watered treatments in grapevine shoots and roots, respectively. The ABA biosynthesis genes beta-carotene hydroxylase, zeaxanthin epoxidase, and 9-cis-epoxycarotenoid dioxygenases were up-regulated in WD root and WD shoot. A positive enrichment of ABA biosynthesis genes and signaling pathways in WD grapevine roots indicated enhanced root signaling to the shoot. An increased frequency of differentially expressed reactive oxygen species scavenging (ROS) genes were found in the WD shoot. Analyses of hormone signaling genes indicated a strong ABA, auxin, and ethylene network and an ABA, cytokinin, and circadian rhythm network in both WD shoot and WD root.

**Conclusions:**

This work supports previous findings in detached leaf studies suggesting ABA-responsive binding factor 2 (*ABF2*) is a central regulator in ABA signaling in the WD shoot. Likewise, ABF2 may have a key role in *V. riparia* WD shoot and WD root. A role for *ABF3* was indicated only in WD root. WD shoot and WD root hormone expression analysis identified strong ABA, auxin, ethylene, cytokinin, and circadian rhythm signaling networks. These results present the first ABA, cytokinin, and circadian rhythm signaling network in roots under water deficit. These networks point to organ specific regulators that should be explored to further define the communication network from soil to shoot.

**Electronic supplementary material:**

The online version of this article (10.1186/s12870-019-1664-7) contains supplementary material, which is available to authorized users.

## Background

Sustainability of woody temperate fruit crops production has become a major concern in today’s context of continued reduction of arable land and water resources due in part to an increase in global warming. The perennial grapevine is the most widely cultivated woody fruit crop with over 7.6 million hectares and 75 million metric tons production [1]. *Vitis riparia Michx.*, the only grape species native to the upper Midwest region of the United States [2], plays a vital role in the global grape industry through its use in rootstocks, which are used commercially throughout the world as well as in hybrid scion cultivar development. *V. riparia* is noted for its resistance to phylloxera, adaptation to variant soil types, and low temperature tolerance, but is suggested to have limited drought tolerance relative to other grapevine genotypes [3–7]. With the changing climatic conditions, drought is a primary environmental constraint on grapevine growth, development, and sustainability [8–11].

Physiological responses to water deficit have been well studied in grapevine, and recently transcriptional changes have been examined in leaf tissues under a variety of water deficit conditions [12–16]. Rootstock and scion experiments have shown that increased root vigor and hydraulic conductance results in increased leaf area and leaf-area specific transpiration [16]. Root perception of soil water status promotes changes in gene expression and hydraulic conductance promoting ABA biosynthesis and signaling in plants [17–19]. Transcriptomic analysis of leaves and roots of drought tolerant and intolerant rootstocks indicate differences in leaf response are genotype dependent [18]. Signals from the root regulate stomata closure, which restricts water loss and limits carbon assimilation [20–25]. The synthesis of ABA and ethylene in the drying root are indicated as long-distance chemical signals to the shoot [15, 17, 19, 24]. While many studies have addressed grapevine leaf and berry molecular responses under water deficit pointing to the central role of ABA in signaling response [13, 14, 26–33], there has been more limited exploration of root signaling characteristics [17, 18]. However, these studies together indicate key roles for ABA and ethylene in grapevine responses to water deficit. We hypothesize that while ABA signaling plays a key role in the complex signaling between the root and shoot, the signaling networks will also include differences due to organ functional differences. In addition, it is expected that root response to water deficit is dependent on carbohydrate metabolism for osmotic potential changes where the carbohydrates are provided in large part by shoot photosynthetic activity. It is therefore essential to determine both the root signaling of soil water conditions and shoot responses to provide a well-informed genomic knowledge base for improved selection of grapevine rootstock and scion cultivars. The roots are the source of water uptake and signals of soil water conditions; therefore, both root and shoot need to be studied together. In this study, *V. riparia* root and shoot responses to a gradually induced water deficit were evaluated to identify signature tissue gene expression responses. The foundational information on root signaling of water deficit for this important rootstock species will be useful as we hypothesize that there will be differences in the root and green shoot signaling responses. This information will help identify key signaling networks and traits for improving grapevine management practices and selection of new rootstocks [30].

## Methods

### Grapevine material and growth conditions

Potted spur-pruned, ten-year-old *V. riparia* Michx. (U.S. National Plant Germplasm System PI588259) vines were removed from cold storage, root pruned, repotted in fresh medium in 15 L pots, and grown under a long photoperiod (15 h) with 25/20 °C ± 3 °C day/night temperatures and 600 to 1400 μmol m^− 2^ s^− 1^ photosynthetic photon flux in a climate-controlled, un-shaded glass greenhouse (En Tech Control Systems Inc., Montrose, MN) in Brookings, SD, USA (44.3°N). Three shoots were allowed to develop on each grapevine. After 30 d, when the grapevine shoots had reached 12–15 nodes, 36 grapevines were randomized into two groups: water deficit (WD) and well-watered control (C). Five days after randomization, differential water treatments began: WD received no water and C received 2 L of tap water each day, which allowed for gravitational water runoff. Shoot elongation (length and node number) was recorded in nine grapevines from each treatment every other day, starting the day before treatment induction. Physiological measurements occurred at 7 and 14 d of treatment on three separate replicates for each treatment; each separate replicate was composed of three vines.

### Physiological measurements

Stem water potential (Ψ_stem_) was measured with a modified 3005HGPL plant water status console (Soil Moisture Equipment Corp., Santa Barbara, CA) on young, newly expanded leaves (5th fully expanded leaf from the shoot tip) as previously described [31]. The leaves were wrapped in foil and sealed in a plastic bag while still on the grapevine to equilibrate to the stem water potential [34]. The leaves equilibrated for two hours, were excised, and Ψ_stem_ was immediately measured with the pressure chamber. One leaf from each grapevine in each replicate was measured; vines were only used once for these destructive measurements, thus no repeat measures were made.

Net photosynthesis rate (Pn) and stomatal conductance (SC) were measured with a CI-340 portable photosynthesis meter (CID, Inc.) just prior to measuring stem water potential (Ψ_stem_). Gas exchange measurements were taken using leaves on separate shoots, but at the same node position as the leaves used for Ψ_stem_. Photosynthetic measurements were made in one grapevine from each replication for each treatment (WD, C) and time point (7 d, 14 d). The leaf chamber covered 11 cm^2^ of the total leaf area and the meter operated under open conditions. Leaf and air temperature were measured by an additional sensor. A CI-301LA light attachment was used to maintain photosynthetically active radiation (PAR) at ≥2400 μmol m^− 2^ s^− 1^. Fresh weight (FW) measurements were recorded from each grapevine on a 4-node shoot tip and 5 cm root tips for each of the three separate replicates (*n* = 3). The organs were then dried at 60 °C and dry weight (DW) recorded. Percent water content (WC), hydration value ((HV: g H2O/g DW; (WC = (FW-DW)/DW)) and dry matter content (DMC: DW/FW) were calculated. At the same time, root tips (2 cm) and 4-node shoot tips were excised, plunged in liquid nitrogen, and stored at − 80 °C for future RNA extraction.

Statistical significance of treatment, time, and interaction effects of Pn, SC, Ψ_stem_, and WC values were determined using analysis of variance (ANOVA). These measures were conducted using new vines at each time point as water potential and water content were destructive measures. Mean separation of significant factors was determined with a Student-Newman-Keuls test at *p* < 0.05. Growth measurements were analyzed with repeated measure ANOVA as the same vines were measured repeatedly throughout the study.

### RNA extraction

Root and shoot samples from the 14 d C and WD treatments were used for transcriptomic analysis, as this time point showed distinct physiological differences between treatments. Total RNA was extracted from 100 mg of frozen pulverized shoot tips as described by [35]. Total RNA was extracted from multiple root tips (5 cm) for each replicate using the Qiagen RNeasy Midi RNA isolation kit (Qiagen, 75,144) according to manufacturer protocol. DNA was removed using an RNase-Free DNase kit (Qiagen, #79254). RNA quality and quantity of the shoot and root tissue were verified using an Agilent 2100 Bioanalyzer and RNA 6000 nano chip (#5067–1511).

### Real-time PCR

Real-time PCR was used to evaluate expression levels of seven ABA metabolic and signaling genes and a reference gene in root and shoot tissues prior to RNA sequencing. Primers were designed with PrimerQuest (Integrated DNA Technologies, http://www.idtdna.com), using the default parameters for real-time PCR (Additional file 1: Table S1). Candidate genes included zeaxanthin epoxidase (*ABA1;* VIT_07s0031g00620*),* 9-cis-epoxycarotenoid dioxygenase 3 (*NCED3;* VIT_19s0093g00550), protein phosphatase 2CA (*PP2CA;* VIT_13s0019g02200), ABA 8′-hydroxylase 3 (*CYP707A3;* VIT_02s0087g00710), molybdenum cofactor sulfurase (*ABA3, SIR3;* VIT_19s0027g01090), protein phosphatase 2C 16 (*ABI1;* VIT_11s0016g03180), and UDP-glycosoyltransferase 73B3 (*UGT73B3*; VIT_03s0063g00040). *V. riparia* eukaryotic initiation factor4A (*VreIF4A*) was used as a reference gene [35] (transcript abundance of this gene was not affected by the water deficit treatment in our RNAseq dataset). Three separate PCR reactions were conducted for each of the three replicates from each treatment. Primer optimization, cDNA synthesis, standard curves, and real-time PCR reactions (including parameters) were conducted as described by [35]. Data analysis was performed with MxProQPCR software (Stratagene, LaJolla, CA, USA). Relative expression ratios of candidate genes (*ABA1, NCED3, PP2CA, CYP707A3*, *ABA3, ABI1,* and *UGT737B3*) to the reference gene (*VreIF4A*) were calculated using R = (E_target_)^ΔCt^_target_^(control-sample)^ / (E_ref_)^ΔCt^_ref_^(control-sample)^; where E = 1 + MxPro efficiency; target = candidate gene; ref. = *VreIF4A*; control = C (well-watered grapevines), [36]. For direct comparison between tissues, all genes were expressed relative to their specific well-watered control. A Student’s t-test, *p*-value ≤0.05 was used to determine significant gene expression differences between root and shoot tissue for individual genes.

### RNA sequencing and analysis

RNAseq was performed at Cornell University Life Sciences Core Laboratories (Ithaca, NY, USA). Bar coded libraries were prepared for three separate replicates of each treatment and organ (root or shoot; two tissues, two water treatments with three replicates for each =12 separate libraries) and they were sequenced using an Illumina® HiSeq™ 2000 Sequencing System (Illumina, Inc., San Diego, CA, USA). Illumina sequences from each of WD and C treatments for each organ (root and shoot) were generated as 100 bp single-end reads in FASTQ format. Quality of sequences were explored with FASTX toolkit and sequences cleaned using Prinseq [37]. The cleaning procedure included, trimming low quality reads from the ends to a Phred quality score > 20 and filtering reads with a length less than 20 bp. Samples after cleaning had high quality reads (20 to100 bp).

Bowtie2 V2.1.0 was used to build the index of the grapevine reference genome assembly (PN40024 12X, V1), a nearly homozygous inbred of the *V. vinifera* Pinot Noir cultivar. TopHat V2.0.8 was used to map each of the cleaned samples to the Bowtie build index and Cufflinks V2.1.1 was used to quantify transcript abundance in terms of Reads Per Kilobase of exon model per Million mapped reads (RPKM). SAMtools V0.1.18 was used for sequence manipulation and the htseq-count script in the HTSeq package was used to count reads mapped to the grapevine gene models. Differential gene expression of the WD treatment relative to the C was determined using the Cuffdiff program within Cufflinks with default parameters. In significantly differentially expressed genes (DEGs) at a false discovery rate (FDR)-adjusted *P*-value (q-value) < 0.05, the terms up- or down-regulated will be used to refer to the expression values of the WD root relative to C root expression values or WD shoot expression values relative to C shoot expression values. The datasets generated for this study can be found in the NCBI sequence read archive under accession #GSE109065, SRA SRP130959.

### VitisNet pathway and gene set enrichment analysis

Gene Set Enrichment Analysis (GSEA) was conducted using read count data from each of the three replicates for each treatment using GSEA-P 2.0 (http://www.broad.mit.edu/GSEA) and 247 VitisNet pathways (https://openprairie.sdstate.edu/vitisnet-12x_files/) including at least 7 genes [38–42]. The recommended GSEA-P 2.0 default parameters of 1000 permutations, nominal *p*-value < 0.05 and FDR q-value < 0.25 were used to identify enriched molecular pathways related to WD or C [39]. Positive significantly enriched pathways are generally up-regulated in WD root or shoot relative to their specific C organ and negatively enriched pathways are down-regulated in WD relative to the respective C organ.

### Network construction for hormone DEGs in root and shoot

The expression values of DEGs that belong to target functions (i.e., ABA, auxin, circadian rhythm, cytokinin, and ethylene) were extracted from the replicated root or shoot gene expression matrix. Then the Pearson correlation coefficients (PCCs) between genes were calculated in R. The PCCs between a gene and itself were assigned zero, as self-correlation was not considered in this study. Next, the correlation matrix was modeled as a network using the *graph_from_adjacency_matrix* function in the igraph R package (https://www.r-project.org/), in which nodes representing genes and weight of edges connecting two genes being the PCCs between the two genes. Only the edges with an absolute weight greater than cutoff for a network were kept in our analysis (ABA, cytokinin, and circadian rhythm networks (0.8 for root and 0.96 for shoot) and ABA, auxin, and ethylene networks (0.96 for root and shoot)). The four generated networks (two each for roots and shoots) were exported as the GML-format files. These files were visualized using Cytoscape (version 3.4.0; www.cytoscape.org) with node size representing degree, edge width reflecting correlation, and node colors to distinguish hormone subnetworks. The topology of these networks follows the *Group Attributes Layout* option in Cytoscape.

## Results

### Water deficit reduced shoot elongation and photosynthesis

WD grapevines had a lower stem water potential (Ψ_stem_) than the controls (Table 1) and there was a strong correlation (r = 0.90) between Ψ_stem_ and photosynthesis (Pn). The Pn and stomatal conductance (SC) were significantly lower in WD grapevines relative to the control grapevines.

**Table 1:** Leaf physiological changes in response to water deficit

Treatment water status	Treatment day	Pn (z)	SC	Ψ_stem_ (z)
		mmol m^− 2^ s^− 1^	mmol m^− 2^ s^− 1^	MPa
C	7	6.76	@	− 0.39
C	14	4.99	14.98	−0.34
WD	7	1.72	@	−1.02
WD	14	1.83	0.57	−1.27

Mean ± SE of net photosynthesis rate (Pn), stomatal conductance (SC) and stem water potential (Ψ_stem_). Two-way ANOVA significant main effects for treatment is noted with z, (*p* ≤ 0.05, *n* = 3); there were no significant time or time x treatment interactions, @ indicates not measured

**Fig. 1:**
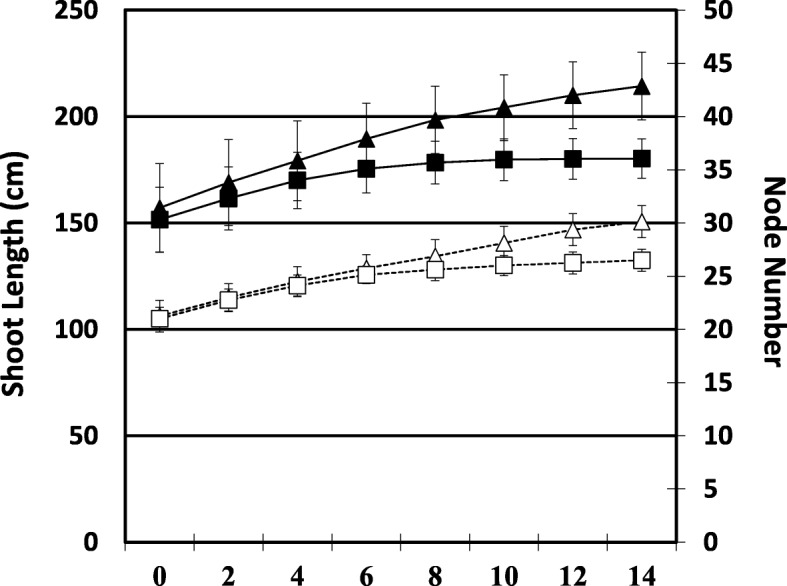
Primary shoot length and node number. Water deficit (WD, squares) and control (C, triangle). Solid lines indicate primary shoot length; dashed lines represent node number

**Fig. 2:**
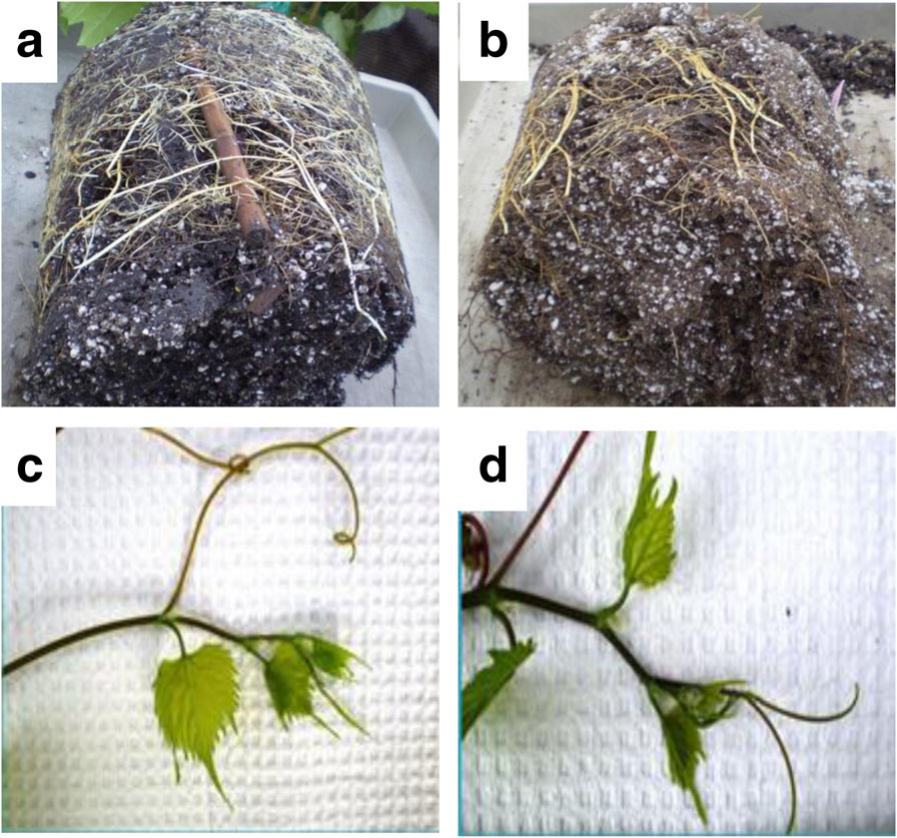
*V. riparia* root ball and shoot tip. **a** Well-watered control root; **b** Water deficit root; **c** Well-watered control shoot tip; **d** Water deficit shoot tip

Shoot elongation and node number were significantly reduced in the WD grapevines in comparison to the control grapevines after 10 d (Fig. 1). Water deficit effects were significant for percent water content (%WC) and dry matter content in shoot tip and root; however, there was not a significant interaction between time and treatment (Additional file 2: Table S2). A small amount of leaf shedding from the base of the WD shoot was observed after 14 d of water deficit. Visual and physical root changes were observed at 14 d of water deficit. Roots were brown, stiffer (not easily bent), and there were fewer roots visible on the exterior of the root ball for the WD grapevines compared to C grapevines (Fig. 2a-b). Shoot tips were similar between both treatments (Fig. 2c-d).

### Distinct root and shoot transcriptome profiles were revealed under water deficit treatment

DEGs in WD roots or WD shoots were further identified as up- or down-regulation relative to their respective C treatment. There were fewer DEGs in WD root (1385) than in the WD shoot (6925) tissues (Additional file 3: Table S3, Additional file 4: Table S4). In the WD root there were 885 up-regulated and 500 down-regulated DEGs, whereas in the WD shoot there was a more similar number of up- and down-regulated DEGs (2978 and 3947, respectively) (Additional file 3: Table S3, Additional file 4: Table S4). Between the root and shoot there were 836 DEGs in common ((Additional file 3: Table S3, Additional file 4: Table S4); DEGs in both organs are in bold in the tables). The majority of the DEGs in common had a similar up-regulated pattern in WD root (560) and WD shoot (522), with only 73 that had opposite regulation in WD root and WD shoot (Additional file 5: Figure S1).

Seven candidate genes (*ABA1, NCED3, PP2CA, CYP707A3*, *ABA3, ABI1,* and *UGT737B3*) in the ABA biosynthesis and catabolism pathways examined by real time PCR were expressed as a fold-change of WD/C of the respective organ (Additional file 6: Figure S2). The UDP-glycosyltransferase (*UGT737B3,* VIT_03s0063g00040) selected was similar to an *Arabidopsis* UDP-glycosyltransferase ortholog, that is specifically expressed in *Arabidopsis* roots (At4g34135); however, it was not expressed in *V. riparia* roots in PCR reactions and not significant in RNAseq analysis. *NCED3* showed significantly greater transcript abundance in WD roots than in WD shoots (*p*-value =0.003) (Additional file 6: Figure S2). In contrast, *CYP707A3* had significantly greater fold-change in WD shoot (p-value = 0.019). There was no difference in the WD/C for *ABA1, PP2CA, ABA3,* and *ABI1* by PCR analysis between root and shoot*.* A strong correlation (an average of r = 0.99) was observed for the expression fold-change of WD to C with real-time PCR and RNAseq expression data. The greater sensitivity of RNAseq analysis was apparent, with the detection of the significant up-regulation of *PP2CA* (VIT_13s0019g02200) and *ABI1* (VIT_11s0016g03180) in WD root and WD shoot (Additional file 3: Table S3, Additional file 4: Table S4) which was not detected by PCR.

### A greater number of molecular pathways were significantly enriched in WD shoot than in WD root

Gene set enrichment analysis using 247 VitisNet pathways indicated there were 25 and 50 VitisNet pathways significantly enriched (FDR q-value < 0.25) in WD root and WD shoot, respectively (Table 2). There were eight VitisNet pathways positively enriched in both WD root and WD shoot (galactose and cyano-amino acid metabolism, photosynthesis, ABA biosynthesis, cytokinin signaling, autophagy, and transcription factors (no apical meristem/ataf1/2/cup-shaped cotyledon (*NAC*) and other zinc finger-an1 (*ZF-AN1*)). Five pathways were negatively enriched in both WD root and WD shoot (nucleotide sugar metabolism, fatty acid biosynthesis, gibberellin signaling, phagosome, and transcription factor (ORPHAN CCT)).

**Table 2:** VitisNet pathway enrichment in water deficit root and shoot

Category	VitisNet pathway	Pathway size	Root		Shoot	
			NES	FDR q-val	NES	FDR q-val
1.1 Carbohydrate Metabolism	**VV10052 Galactose Metabolism**	**149**	**1.59**	**0.17**	**1.54**	**0.18**
	**VV10520 Nucleotide Sugars Metabolism**	**61**	**−1.48**	**0.30**	**−1.88**	**0.01**
	VV10530 Aminosugars Metabolism	79	1.10	0.90	−1.64	0.09
1.2 Energy Metabolism	**VV10195 Photosynthesis**	**114**	**1.74**	**0.09**	**1.13**	**0.47**
	VV10196 Photosynthesis Antenna Proteins	19	1.99	0.00	−0.86	1.00
	**VV10680 Methane Metabolism**	**113**	1.56	0.17	−1.60	0.11
1.3 Lipid Metabolism	**VV10061 Fatty Acid Biosynthesis**	**70**	**−1.83**	**0.09**	**−1.64**	**0.09**
	VV10561 Glycerolipid Metabolism	144	1.09	0.91	1.55	0.20
1.5 Amino Acid Metabolism	VV10360 Tyrosine Metabolism	147	1.12	0.88	−1.46	0.23
	VV10400 Phenylalanine Metabolism	200	0.95	0.97	−1.71	0.06
	VV10400 Phenylalanine Tyrosine & Tryptophan Biosynthesis	143	1.12	0.88	−1.55	0.16
1.6 Other Amino Acid Metabolism	**VV10460 Cyanoamino Acid Metabolism**	**34**	**1.60**	**0.16**	**2.00**	**0.02**
	VV10480 Glutathione Metabolism	131	1.26	0.58	2.23	0.00
1.7 Glycan Biosynthesis & Metabolism	VV10511N-Glycan Degradation	65	0.85	1.00	−1.67	0.08
1.8 Cofactors & Vitamin Metabolism	VV10790 Folate Biosynthesis	30	1.24	0.62	1.56	0.20
	VV10860 Porphyrin and Chlorophyll Metabolism	64	1.60	0.17	1.18	0.52
1.9 Biosynthesis of Secondary Metabolites	VV10900 Terpenoid Biosynthesis	156	0.93	0.99	−1.53	0.18
	VV10902 Monoterpenoid Biosynthesis	181	1.46	0.29	1.61	0.18
	VV10904 Diterpenoid Biosynthesis	68	−1.18	0.72	−1.45	0.23
	VV10906 Carotenoid Biosynthesis	41	1.29	0.52	2.05	0.02
	VV10940 Phenylpropanoid Biosynthesis	222	0.68	1.00	−1.80	0.03
	VV11002 Auxin Biosynthesis	90	1.20	0.69	1.58	0.20
	**VV11013 ABA Biosynthesis**	**16**	**1.88**	**0.02**	**1.98**	**0.02**
1.10 Other Metabolism	**VV11000 Single Reactions**	**167**	**1.54**	**0.18**	**−1.57**	**0.14**
2.4 Replication and Repair	VV23030 DNA Replication	64	−1.40	0.36	−1.63	0.09
3.2 Hormone Signaling	VV30003 ABA Signaling	151	1.61	0.17	1.15	0.53
	VV30007 Auxin Signaling	271	0.98	0.97	−2.03	0.00
	**VV30007 Cytokinin Signaling**	**68**	**1.56**	**0.19**	**1.46**	**0.25**
	**VV30010 Gibberellin Signaling**	**36**	**−1.93**	**0.07**	**−1.61**	**0.11**
3.3 Plant-specific Signaling	VV34627 R Proteins from Plant Pathogen Interaction	349	0.71	1.00	−2.03	0.00
	Circadian Rhythm	64	1.58	0.17	1.15	0.52
4.1 Transport and Catabolism	VV44140 Regulation of Autophagy	**26**	**1.56**	**0.16**	**1.55**	**0.18**
	**VV44145 Phagosome**	**118**	**−1.80**	**0.09**	**−1.45**	**0.23**
4.2 Cell Motility	VV44180 Regulation of Actin Cytoskeleton	343	−0.96	1.00	−2.07	0.00
4.3 Cell Growth and Death	VV40006 Cell Wall	454	1.05	0.94	−1.72	0.07
	VV44110 Cell Cycle	323	−0.96	1.00	−2.16	0.00
5.1 Membrane Transport	VV52010 ABC Transporters	239	0.98	0.98	1.49	0.22
5.2 Hormone Transport	VV50004 Auxin Transport	52	1.13	0.86	−1.69	0.07
5.3 Transport System	VV50113 Thylakoid Targeting Pathway	48	1.72	0.07	−1.22	0.49
5.4 Transporter Catalog	VV50105 Transport Electron Carriers	61	1.74	0.07	−0.65	1.00
	VV50122 Porters Cat 7 to 17	237	1.32	0.51	−1.44	0.23
	VV50135 Primary Active Transporter Cat D2 to E2	81	1.16	0.80	1.84	0.04
6.0 Transcription Factors	VV60003 AP2 EREBP	137	1.34	0.50	−1.49	0.20
	VV60004 ARF	29	0.79	1.00	−1.48	0.21
	VV60008 AUXIAA	23	1.00	0.97	−1.68	0.07
	VV60011 BHLH	148	1.24	0.61	−1.72	0.07
	VV60015 C2C2-DOF	25	1.63	0.16	−1.05	0.74
	VV60019 C2C2-YABBY	7	1.72	0.08	−1.27	0.46
	VV60022 CPP	7	1.21	0.66	−1.49	0.20
	VV60029 G2-LIKE	37	1.54	0.18	1.30	0.37
	VV60033 GRF	12	−0.74	1.00	−1.50	0.19
	VV60044 MYB	166	0.96	0.97	−1.51	0.19
	**VV60046 NAC**	**75**	**1.65**	**0.14**	**1.88**	**0.04**
	VV60055 SBP	20	1.26	0.57	−1.51	0.19
	VV60061 TCP	19	0.89	1.00	−1.52	0.19
	**VV60070 ORPHANS CCT**	**8**	**−1.77**	**0.08**	**−1.44**	**0.24**
	VV60073 ORPHANS ZF-B BOX	15	1.27	0.55	1.87	0.041
	**VV60077 OTHER ZF-AN1**	**13**	**1.53**	**0.19**	**1.74**	**0.08**
	VV60079 OTHER ZF-DHHC	23	−0.91	1.00	−1.48	0.21
	VV60082 GNAT	36	1.56	0.18	−0.89	0.99

A positive normalized enrichment score (NES) indicates VitisNet pathways enriched in WD tissues and a negative NES indicates pathways enriched in C treatments at nominal *p*-value < 0.05 and false discovery rate (FDR) q-value < 25%. Bold indicates an enrichment in both root and shoot

Positively enriched pathways specific to WD root were ABA and circadian rhythm signaling, photosynthesis antenna proteins, porphyrin and chlorophyll metabolism, transport (thylakoid and electron carriers), and transcription factors (C2C2-dna-binding with one finger (*C2C2-DOF)*, *C2C2-YABBY*, *G2-Like*, and gcn5-related n-acetyltransferases (*GNAT*)) (Table 2). A total of nine pathways (glycerol lipid and glutathione metabolism; folate, monoterpenoid, carotenoid, and auxin biosynthesis; ATP-binding cassette transporters (*abc transporter*) and primary active transporter; and orphan zinc finger-b box transcription factors) were positively enriched only in WD shoot. Conversely, canonic shoot pathways related to photosynthesis were enriched in root and not in the shoot. In addition, only two carbohydrate metabolism pathways were positively enriched in WD root and negatively enriched in WD shoot (methane and single reactions). A large number of pathways (26) were negatively enriched only in WD shoot, including pathways in carbohydrate and amino acid metabolism, secondary metabolite biosynthesis, DNA replication, cell wall and cycle, auxin transport, and multiple transcription factor families.

### ABA biosynthesis and catabolism pathways had similar expression profiles in WD root and WD shoot

Two beta-carotene 3-hydroxylase 1 genes (VIT_02s0025g00240, VIT_16s0050g01090) upstream of ABA biosynthesis pathway were up-regulated in both WD root and WD shoot (Additional file 3: Table S3, Additional file 4: Table S4). In the first committed step of the ABA biosynthesis pathway, two differentially expressed *NCED* genes (VIT_10s0003g03750, VIT_19s0093g00550) were up-regulated in WD root and only the latter was differentially up-regulated in WD shoot. Abscisic acid aldehyde oxidase was not differentially expressed in either organ. UPD-glycosyltransferase (VIT_12s0055g00020), which promotes ABA conjugation to ABA-GE [43], was up-regulated in WD shoot. This can be reversed by beta glucosidase (VIT_01s0011g00760, VIT_17s0000g02680) which were up- and down-regulated respectively in WD shoot. Degradation of ABA to phaseic acid is initiated by ABA 8′-hydroxylase (*ABAHASE*). Three putative ABAHASE genes (VIT_06s0004g05050, VIT_03s0063g00380, and VIT_02s0087g00710) were differentially up-regulated in WD shoot and/or WD root.

### A greater number of up-regulated ABA, cytokinin, and circadian rhythm signaling DEGs were found in WD shoot than in WD root

In WD roots, there were more auxin, ABA, cytokinin, ethylene, jasmonate, and circadian rhythm DEGs up-regulated than down-regulated. WD shoot had greater numbers of hormone related DEGs than WD root; however, more auxin and ethylene related DEGs were down-regulated rather than up-regulated.

**Fig. 3:**
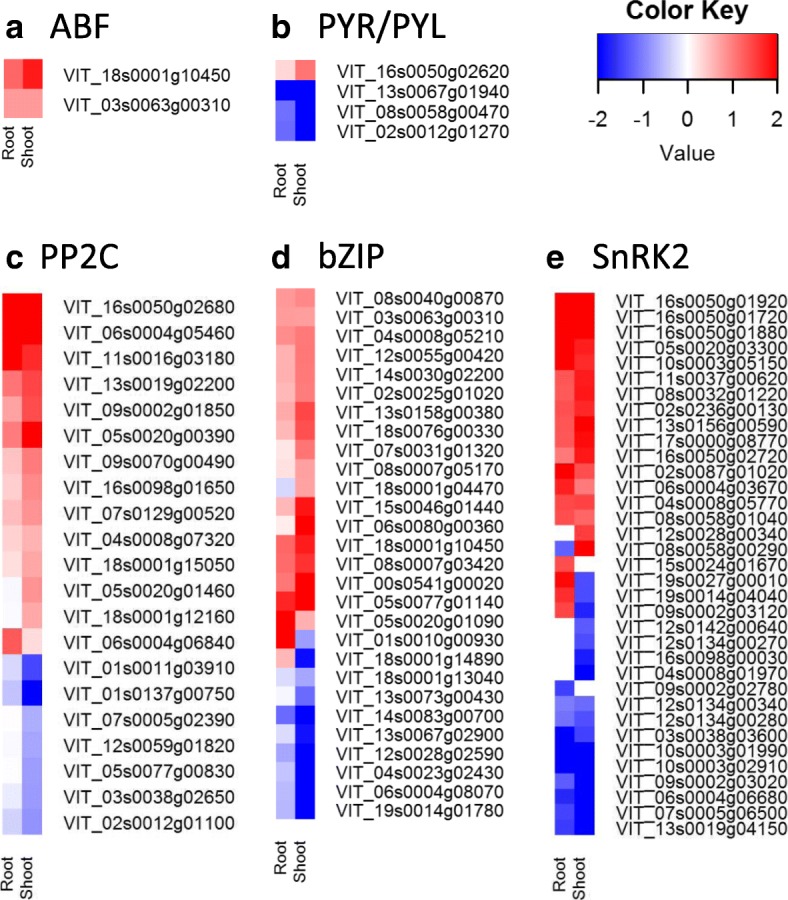
ABA biosynthesis and catabolism related gene expression profiles. **a** ABF; **b** PYR/PYL; **c** PP2C; **d** bZIP and **e** SnRK2. Heat map values are fold change log_2_, the red and blue colors represent up- and down-regulation of the gene expression in water deficit tissue relative to their respect to control tissue in root and shoot

In WD grapevine, a greater number of ABA signaling DEGs were found in WD shoot (35, 26) than in WD root (20, 4) (up-, down-regulated, respectively). The ABA signaling genes pyrabactin resistance1)/PYR1-like/regulatory components of aba receptors (*PYR/PYL/RCAR*) were down-regulated in WD root and shoot with the exception of *PYL6* (VIT_16s0050g02620) in WD shoot (Fig. 3). In WD root, all protein phosphatase type 2C (*PP2C*) DEGs (5) were up-regulated, and in WD shoot, 13 of the 20 DEGs were up-regulated. A similar number of the serine/threonine kinases (*SnRK2*) DEGs were up-regulated in WD root (14) and WD shoot (15) and there were two *SnRKs* DEGs (VIT_15s0024g01670 and VIT_09s0002g03120) up-regulated in the WD root. The *SnRKs* phosphorylate a group of ABA-responsive element binding factors (*abf*s, basic leucine zipper (*bZIP*) transcription factors) which activate downstream gene expression. In both the WD shoot and WD root, downstream genes *ABF*2 and *ABF3* were up-regulated relative to its respective C grapevine organ. Several other *bZIP* transcription factor genes were differentially expressed (Fig. 3); however, only two were up-regulated in both WD root and WD shoot (abscisic acid insensitive 1 and 5 (*ABI1, ABI5*)).

### Ethylene response transcription factors were up-regulated in root and shoot

Ethylene signaling has been associated with water deficit in leaves [14] and in this study there were 83 DEGs out of the total 213 annotated as ethylene signaling genes [42], (34 up- and 49 down-regulated). Ethylene and auxin DEGs had a greater number of down-regulated genes than up-regulated genes in WD shoot. In WD root, 21 of 27 DEGs, predominately ethylene response transcription factors (*ERF*), were up-regulated. Of these only 14 were up-regulated in common between the WD root and WD shoot. Auxin and gibberellin signaling pathways were negatively enriched in WD root and WD shoot. In WD shoot, there were 165 DEGs out of the 467 total annotated auxin biosynthesis, signaling and transporter related genes [42], and 35 of these were up-regulated (predominately signaling genes, 21 total). In the WD root, there were only 29 auxin-related DEGs and 18 were up-regulated (15 signaling and 3 biosynthesis genes). Ten of these were up-regulated in common with those in WD shoot. In contrast to WD shoot, there were more auxin and ethylene DEGs up-regulated in WD root than were down-regulated.

### The cytokinin signaling pathway was positively enriched in water deficit shoots

The cytokinin signaling pathway was positively enriched in both WD root and WD shoot. In WD shoot, there were 30 DEGs of the total 80 cytokinin signaling pathway genes with multiple type-A arabidopsis response regulators (*ARRs,* e.g. 5) and arabidopsis psuedo response regulators (*APRR*, e.g. 4) up-regulated in WD shoot (Additional file 3: Table S3, Additional file 4: Table S4). The majority of these were up-regulated (20) and involved in regulation of transcription. In contrast, only four genes were up-regulated in WD root and two of these were in common with the WD shoot. Of the 79 circadian rhythm pathway genes, there were a similar number of circadian rhythm DEGs up-regulated in WD root (10) and WD shoot (15) (Additional file 3: Table S3, Additional file 4: Table S4), with five of these DEGs in both WD shoot and WD root. WD root had only one down-regulated circadian rhythm DEGs (constans-like 14) and all other constans-like DEGs were up-regulated in both WD shoot and WD root.

### ABA, cytokinin, and circadian rhythm networks identified in water deficit root and shoot

**Fig. 4:**
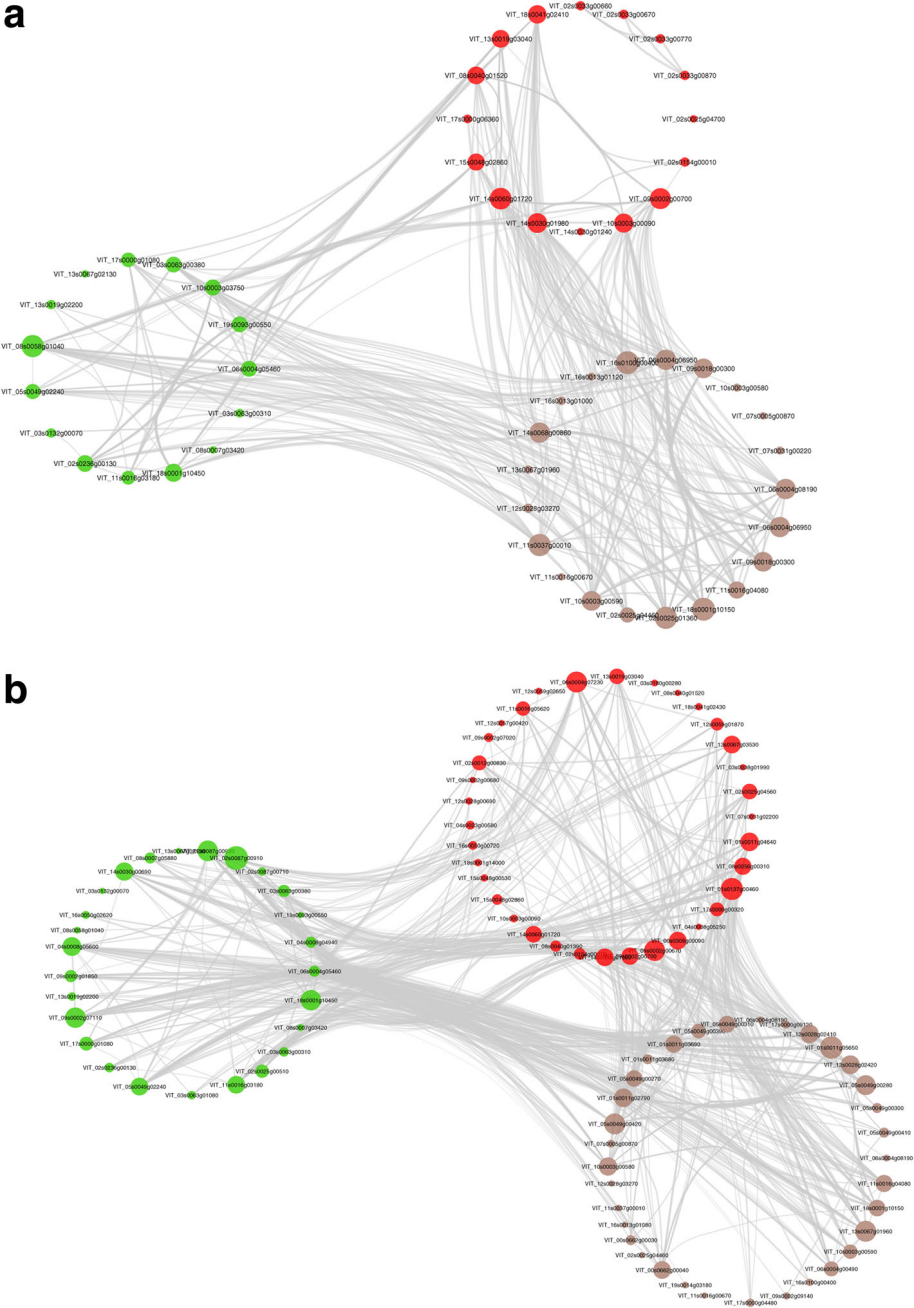
Root and shoot ABA, auxin, and ethylene signaling networks. **a** root network; **b** shoot network. The differentially expressed gene correlation matrix was modeled using the *graph from adjacency matrix* function in igraph R package (ABA = light green, auxin = red, ethylene = grey-brown). Network gene list and annotation can be found in Additional file 7: Table S5a, b

**Fig. 5:**
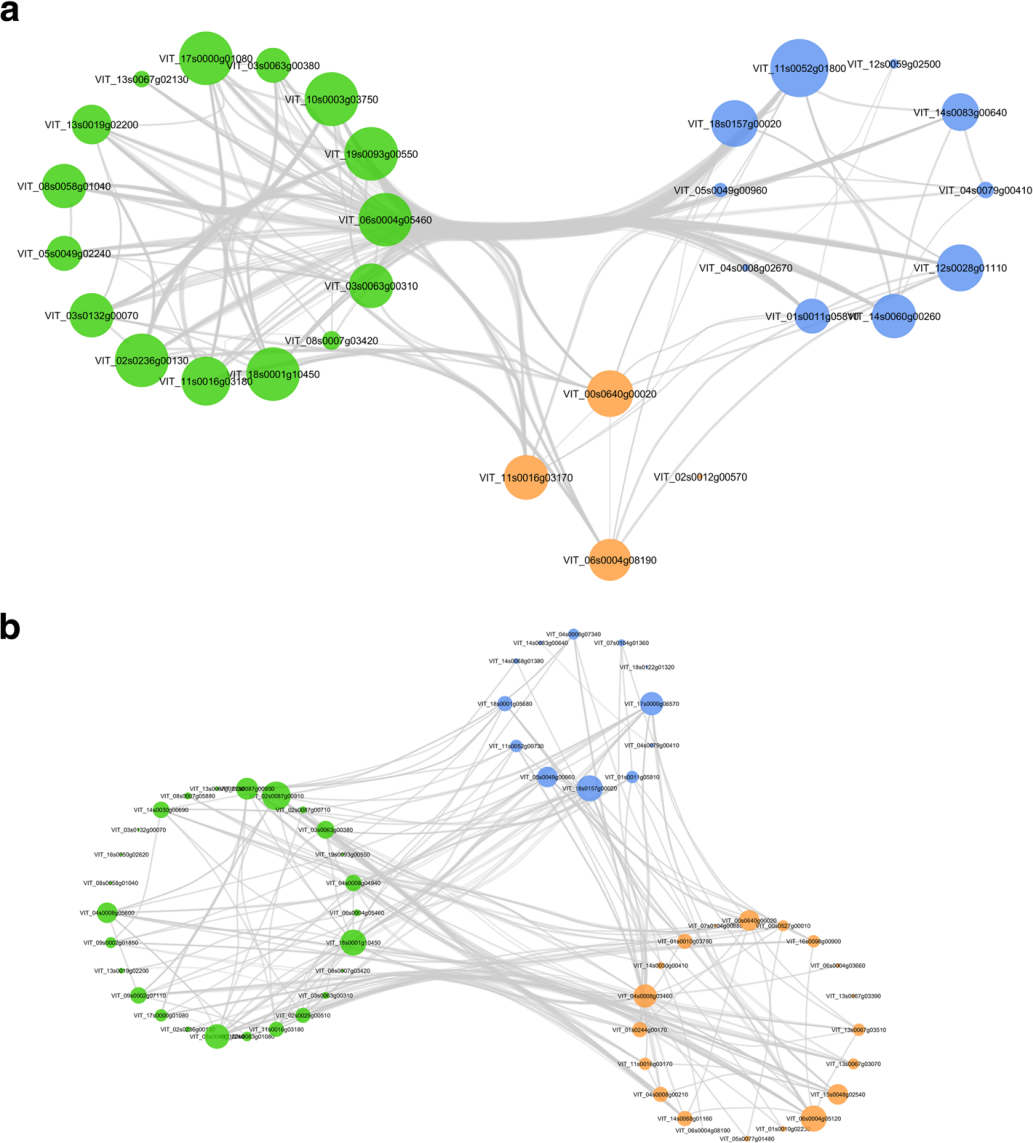
Root and shoot ABA, cytokinin, and circadian rhythm signaling network. **a** root network; **b** shoot network. The differentially expressed gene correlation matrix was modeled using the *graph from adjacency matrix* function in igraph R package (ABA = light green, cytokinin = blue, circadian rhythm = orange). Network gene list are found in Additional file 7: Table S5c, d

Networks were constructed for ABA, auxin, and ethylene or ABA, cytokinin, and circadian rhythm related DEGs. There was a greater number of DEGs with a high correlation between ABA and ethylene than between ABA and auxin in both root and shoot (Fig. 4a, b). *ABF2* is strongly correlated with ethylene in both the root and shoot, whereas *ABF3* is only correlated in the root. The ABA, cytokinin, and circadian rhythm had a strong correlation with ABA signaling genes (interaction, in particular *ABF2*, *ABF3, SnRK2,* cytokinin response factor (*CRF4*) Arabidopsis histidine phosphotransferase (*AHP4*), gigantea (*GI*)*,* constans-like (*COL3*), and phytochrome interacting factor 4 (Fig. 5a). In WD shoot, strong correlations between ABA and cytokinin signaling genes also showed similar correlation with the circadian rhythm genes: *ABF2,* histidine kinase 1 (*AHK3*), and Arabidopsis type b cytokinin response regulators (*ARR1*) signaling genes were correlated with *GI* and timing of cab expression 1 protein (*TOC1*) (Fig. 5b). A total gene list for each network is included in the Additional file 7: Table S5a-d.

### Aquaporin and ROS scavenging genes had distinctly different expression patterns in water deficit root and shoot

Examination of the VitisNet pathways significantly enriched in WD root or WD shoot indicated that several gene families previously noted to be involved in water deficit responses were not only differentially expressed relative to their control but showed different response profiles in root and shoot. Major differences were observed in aquaporin and reactive oxygen species scavenging (ROS) DEGs. It is noteworthy that there were only two up-regulated aquaporins in WD roots (VIT_08s0040g018 90, VIT_14S0108g00700) and 16 aquaporin DEGs that were all down-regulated in WD shoot (Additional file 3: Table S3, Additional file 4: Table S4).

**Fig. 6:**
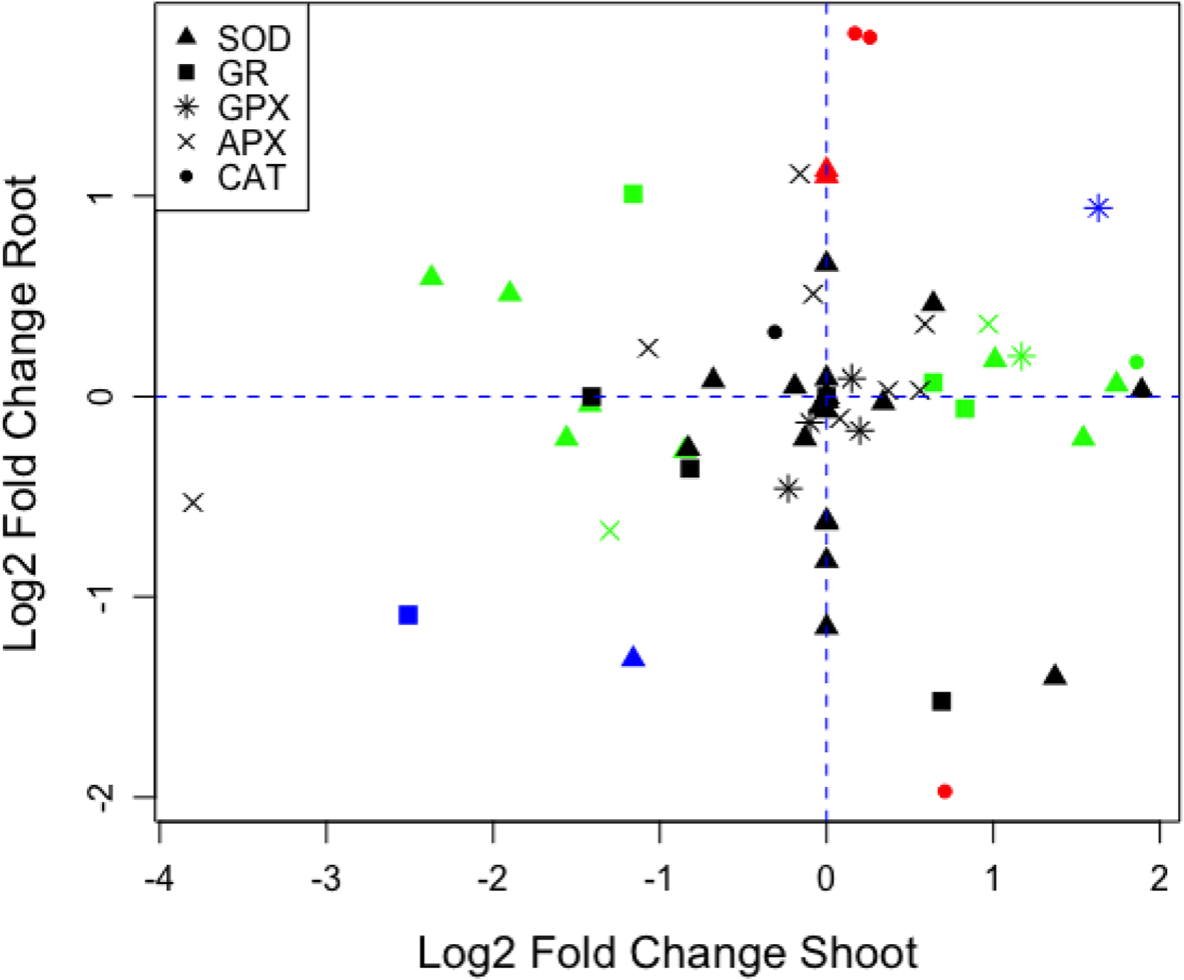
Scatter plot of fold change of genes associated with reactive oxygen species (ROS) scavenging enzymes. ROS scavenging gene families are identified in the legend at top right and differentially expressed (q-value < 0.05). Superoxide dismutase (SOD, triangle), glutathione reductase (GR, square), glutathione peroxidase (GPX, star), ascorbate peroxidase (APX, x) and catalase (CAT, circle) related genes are color coded for shoot (green) and root (red). Genes right and left of the vertical dash line represent up- and down- regulated, respectively in water deficit (WD) relative to well-watered control (C) shoot (green). Genes above and below the horizontal line represent up- and down- regulated respectively in WD root relative to C root (red). Markers for gene family members differentially expressed in both root and shoot are blue and markers for genes not differentially expressed are black. A complete list of ROS related genes are found in Additional file 8: Table S6

A greater number of ROS scavenging related genes were differentially expressed in the WD shoot than in the WD root relative to their respective C (Fig. 6, Additional file 8: Table S6). In WD shoot, six of the 36 genes responsible for synthesizing superoxide dismutase (*SOD*) were down-regulated and three were up-regulated relative to C shoot. Only two SOD genes were up-regulated in WD root and one (VIT_14s0060g00120) was down-regulated in common with WD shoot. Many more genes in the glutathione cycle were up-regulated in the WD shoot (22) than in the WD root (11). Four glutathione reductase (*GR*) genes were differentially expressed in WD shoot, with one of the down-regulated in common with WD root. Genes encoding glutathione peroxidases (*GPX:* VIT_02s0025g03600, VIT_04s0008g06780) were up-regulated in WD shoot, with one in common with WD root. Two ascorbate peroxidase (*APX*) genes were up-regulated in WD shoot and none were differentially expressed in WD root. In contrast, three CATALASE (*CAT*) genes were down-regulated in WD root and one up-regulated in WD shoot. The phenylpropanoid pathway is a source of other potential antioxidant compounds (lignins, stilbenes, flavonoids, and anthocyanins). The WD shoots had a greater number of up-regulated phenylpropanoid biosynthesis DEGs than WD roots. Some genes in the flavonoid synthesis pathway were up-regulated, but more were down-regulated including stilbene synthases.

### Different transcription factors profiles were associated with WD in root and shoot

**Fig. 7:**
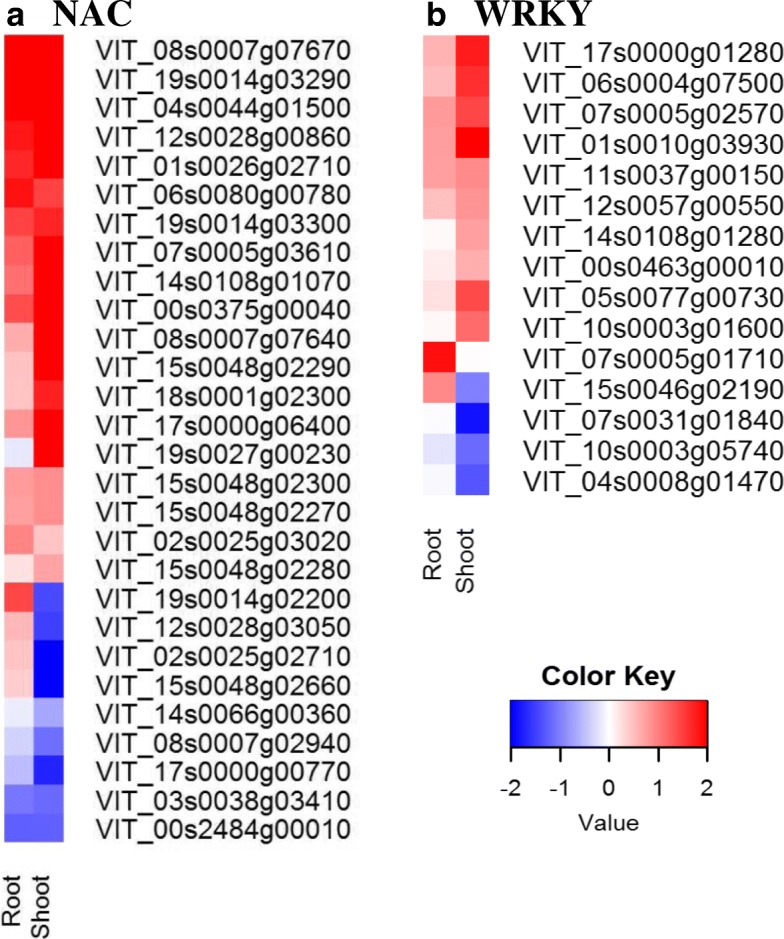
*NAC* and *WRKY* transcription factor differential gene expression (DEG) profile in water deficit (WD) root and shoot tissue. **a** *NAC* transcription factor DEG. **b** WRKY transcription factor DEG. The red and blue colors represent up- and down-regulation of the gene expression in water deficit tissue relative to their respect to control tissue in root or shoot. Expression values are expressed fold change WD/C (log_2_)

**Fig. 8:**
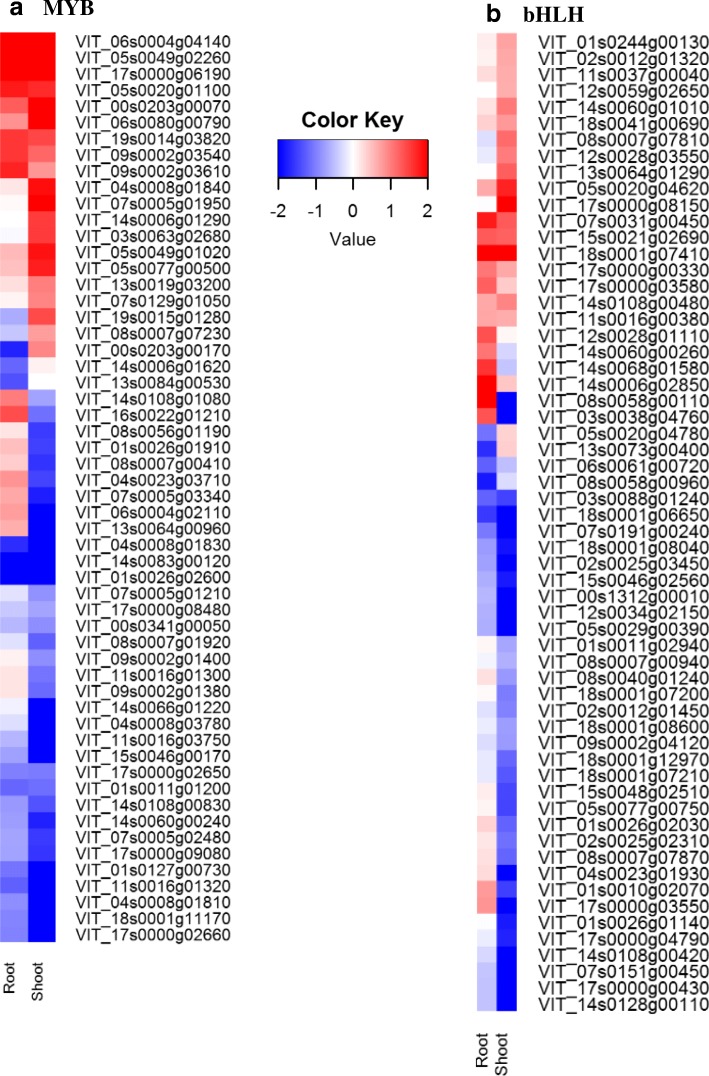
*MYB* and *bHLH* transcription differential gene expression (DEG) profile in water deficit (WD) root and shoot tissue. **a** *MYB* transcription factor DEG. **b** *bHLH* transcription factor DEG. The red and blue colors represent up- and down-regulation of the gene expression in water deficit tissue relative to their respect to control tissue in root or shoot. Expression values are expressed fold change WD/C (log_2_)

There were 62 transcription factors up-regulated and ten down-regulated in common between WD root and WD shoot, and eight that were differentially regulated (Additional file 3: Table S3, Additional file 4: Table S4). A large transcriptional regulatory activity potential was indicated in the WD shoot. A large number of transcription factor DEGs (220 up-regulated, 264 down-regulated) were expressed only in the WD shoot; in contrast, 42 DEGs were expressed only in WD root (Additional file 3: Table S3, Additional file 4: Table S4). The transcription factors (*NAC*, *C2C2-DOF*, *C2C2-YABBY*, *G2-like*, other *ZF-AN1,* and *GNAT*), which have been associated with drought tolerance in grapevine [14, 18] were significantly enriched in WD root (Additional file 3: Table S3). A greater number of *NAC* (Fig. 7a) DEGs were up-regulated in WD shoot (17) than WD root (9). There were 8 *NAC* DEGs in common between the WD organs (Additional file 3: Table S3, Additional file 4: Table S4). The majority of the *WRKY* DEGs in the WD shoot were up-regulated (Fig. 7b) and there was only one up-regulated WRKY transcription factor DEG in WD root (*WRKY23,* VIT_07s0005g01710). A larger number of *MYB*s, which target genes that are involved in the biosynthesis of phytohormones and cell walls, were differentially expressed in WD shoot (71, 32) in contrast to WD root (18, 9) (total, up-regulated respectively) (Fig. 8a). It should be noted that 15 of these were DEGs in both WD organs. A similar pattern was observed in the basic helix loop helix (*bHLH*), transcriptional activators in ABA signaling; however, few were in common with *bHLHs* DEGs in WD root (Fig. 8b). There were few dehydration responsive element binding (*DREB*) transcription factors expressed in WD shoot, and only one in WD root. The bZIP transcription factors that are regulators for the ABA mediated abiotic stress signaling pathways, were up-regulated in WD root and up- and down-regulated in WD shoot.

## Discussion

This study provided a physiological and genome-wide transcriptomic analyses of water deficit responses of shoots and root in *V. riparia,* a species commonly used as a rootstock and in breeding rootstock and scion cultivars. Shoot elongation, photosynthetic and stomatal activity, water status, and morphological characters were observed to target the time point for transcriptomic analysis. *V. riparia* had decreased shoot elongation after 10 d of water deficit, similar to other studies of potted grapevines [44–46]. After 14 d of water deficit, the physiological status of the grapevine characterized by Ψ_stem,_ P_n,_ SC, and WC was significantly impacted in WD grapevines relative to the control grapevines. Leaf shed, cupping, and wilting were observed at 14 d of WD. Leaf shed has been shown to reduce water loss during periods of stress in grapevine [47]. In addition, shedding older leaves allows reallocation of the carbon, nitrogen, and other nutrient resources to support growth in the stem or younger leaves in grapevine and other woody species [20, 48, 49]. Changes were observed visually in the root balls; roots became brown and less flexible, and there were fewer roots visible in WD compared to C grapevines. These characteristics indicate that both root and shoot were adjusting to the water deficit treatment. In this study; however, there were three times as many DEGs in WD shoot than in WD root in strong contrast to a previous WD study in grapevine rootstocks [18]. The difference between the present study and the rootstock study [18] may be due to differences in genotype, WD sensitivity or differences in the length of the water deficit treatment [50].

### Energy related pathways were enriched in WD root

Water deficits in plants cause stomatal closure in the leaves, inhibition of photosynthetic activity and reduction in carbon fixation [51]. Water stress accompanied with heat enhances respiration and methane (CH_4_) emission [52]. In *V. riparia*, photosynthesis and stomatal conductance decreased in the WD treatment. In contrast, VitisNet photosynthesis molecular pathways were positively enriched and genes in both photosystem I and II were upregulated in WD root and WD shoot. A similar decrease in net photosynthesis and up-regulation in photosynthesis related genes and proteins was observed in water-stressed rootstock and scion varieties [13, 27, 53].

Plants alter carbohydrate metabolism equilibrium by accumulating a large amount of water-soluble carbohydrates. In rice, carbohydrate metabolism pathways (fructose and mannose; starch and sucrose metabolism; amino sugars and nucleotide sugars and galactose) are significantly enriched during drought [54]. In contrast, amino sugars and nucleotide sugar metabolism pathways were negatively enriched in WD shoot and WD root in this study. In the woody *Jatropha curcas,* starch, sucrose, and galactose pathways are enriched in response to drought and have the greatest number of DEGs in common to both root and leaves under drought conditions [48]. Similarly, in WD grapevine, galactose metabolism was positively enriched in both WD root and WD shoot. Similar increases in galactose metabolism DEGs and proteins are observed in leaves of rootstocks and Cabernet Sauvignon [14, 27]. Galactose metabolism is important in drought tolerance mechanisms and may provide antioxidant activities [55, 56]. This may also indicate carbohydrate accumulation in grapevine in response to growth suppression under water deficit or down-regulation of sugar transporters [57].

### Cell structure and function pathways were enriched in WD shoot

Actin cytoskeleton mediates cell motility and shape changes during the cell-division cycle and in response to extracellular stimuli [58]. Osmotic stress causes rapid and reversible changes in actin filament organization [59]. In grapevine, the actin cytoskeleton pathway was negatively enriched in WD shoot most likely due to decreased growth under water deficit.

Changes in lipid composition help maintain membrane integrity and preserve cell compartmentation in plants during drought. Leaf lipid content in *Arabidopsis thaliana* decreases progressively under drought [60]. In grapevine, drought tolerant cultivars show an increase in total lipid content in the leaves, in contrast to the drought sensitive cultivars [61]. In *V. riparia*, the fatty acid biosynthesis pathway was negatively enriched in WD root and WD shoot. In contrast, the glycerolipid pathway was upregulated in both the WD organs. This coupled with the up-regulation of the galactose pathway indicates active maintenance of the photosynthetic membranes under water deficit conditions [62].

The autophagy pathway was enriched in both the WD root and WD shoot in *V. riparia*. Autophagy plays a role in protein quality control by targeted degradation of misfolded and damaged proteins induced during biotic and abiotic stresses [63]. Autophagy genes are differentially regulated in roots and shoots of drought-stressed pepper, with a greater up-regulation in roots and stems than in leaves [64]. The heat shock transcription factor A1a (*HsfA1a)* regulates autophagy related genes and overexpression of *HsfA1a* promotes non-ABA dependent drought tolerance and autophagosome formation in tomato [65]. In WD shoot several autophagy genes were up-regulated and phagosome genes were both up- and down-regulated. Several heat shock transcription factors were up-regulated in the WD shoot; however, an ortholog was not found for *HsfA1a.* Further comparative exploration of these genes in drought tolerant and sensitive cultivars may provide greater understanding in grapevine.

### ROS scavenging gene enrichment in WD shoot

In the leaves, stomata closure in response to low shoot water potentials and soil moisture content contributes to reduced photosynthesis and increased respiration [24, 51, 66]. Under low CO_2_ and excess light, reactive oxygen species are formed due to partial reduction or activated derivatives of molecular oxygen from the electron transport activities of chloroplasts, mitochondria, and peroxisomes [67, 68]. Prolonged water stress leads to overproduction of reactive oxygen species that exceeds cellular quenching mechanisms thus disrupting cellular homeostasis [69]. This results in oxidation of proteins, peroxidation of lipids, damage to nucleic acids, inhibition of enzymes, activation of programmed cell death pathway and ultimately cell death [69, 70]. ROS scavenging enzymes are early stress signaling messengers often associated with drought-induced ABA synthesis in water stressed plants [13, 15]. The present study showed a greater number of ROS scavenging genes DEGs particularly in WD shoot, which was similar to increases noted in *V. vinifera* scion and rootstock cultivar leaves during drought stress and may be related to the electron transport activity in leaves [13, 27, 71].

### ABA biosynthesis and ABA signaling had common water deficit transcriptomic responses in roots and shoots

Grapevine leaf dehydration and potted and field water deficit studies indicate a strong role for ABA and other hormone signaling in grapevine acclimation to water stress [13, 14, 17, 18]. Prior to ABA synthesis, carotenoid biosynthesis initiates with the synthesis of the isopentenyl pyrophosphate (*IPP*), and gradually feeds into the ABA biosynthesis pathway [27]. Zeaxanthin epoxidase (*ABA1)* catalyzes an early step in the cascade towards ABA biosynthesis [72]. In *V. riparia*, *ABA1* expression was up-regulated in WD root but down-regulated in WD shoot. Increased activity of *ABA1* in WD root was also observed in tomato and Arabidopsis under water stress [73, 74]. In addition, the *VDE* genes were also up-regulated in WD root but down-regulated in WD shoot. In the ABA metabolic pathway, the first committed step in ABA biosynthesis is initiated by 9-cis-epoxycarotenoid dioxygenase (*NCED*), which converts violaxanthin or neoxanthin to xanthoxin. Two of the three *NCED* genes were up-regulated in WD root, but only one in WD shoot. This result was in agreement with results in rootstocks and scions [17, 18] that show NCED transcripts increase in both shoot and root in response to water deficit; thus indicating a strong role for root ABA signaling to the shoot.

ABA levels can be decreased by ABA catabolism, inactivation, or negative regulation. The ABA 8′-hydroxylase enzyme catabolizes ABA into 8′- hydroxy ABA, which non-enzymatically converts to phaseic acid before reducing to dihydroxyphaseic acid. ABA, in the presence of an ABA-glucosyltransferase enzyme, is metabolized into inactive ABA-glucose ester (ABA-GE). Cleavage of glucose from ABA-glucose ester by β-glucosidases converts ABA-GE back to ABA. Although water stress acts as a signal to transport ABA-GE stored in the vacuole or apoplastic space to the endoplasmic reticulum, its involvement in root-to-shoot signaling is yet to be revealed [29, 75, 76]. Several studies report an increase of *UDP-glycosyltransferase* under stress conditions [77, 78]. ABA-GE concentration increases significantly in WD Chardonnay berries but not in Cabernet Sauvignon [29]. In this study, several UDP-glycosyltransferase genes were up-regulated in the WD shoot, but not in the WD root. Further studies are needed to get a complete picture of how ABA levels are controlled in grapevine.

ABA signaling genes involved in the water stress response include *PYR/PYL/RCAR*, *PP2C* (negative regulator) and *SnRK2* (positive regulator) [28]. The ABA receptor PYR/PYL/RCAR family controls ABA signaling by inhibition of *PP2C* activity [79–82]. Major inhibition of *PP2C* occurs in the presence of ABA; however, the ABA receptors (RCAR/PYR1/PYL) are known to interact with *PP2C*s in a complex manner [83, 84]. *SnRK2*, when activated by ABA, phosphorylates ABA-responsive element binding factors (*ABFs*). In addition, target of rapamycin (*TOR*) kinase, a central regulator of plant metabolism and growth, phosphorylates PYR/PYL/RCAR receptors in unstressed conditions, inactivating the core ABA signaling pathway [85]. Under stressed conditions, ABA stimulated SnRKs, phosphorylate the TOR complex component, *RAPTOR*, providing a mechanism for altering cell metabolism and growth. In *V. vinifera* and hybrid rootstocks, high constitutive expression levels of *PYR/PYL*/*RCAR*s and *SnRKs* are found indicating that the transcript abundance of ABA receptors was not a key component contributing to genotypic differences in water deficit response [17]. However, in a grapevine leaf dehydration study [14], differences in *SnRK 2.6* (*OST1*) are found in gene expression profiles over time, with *V. riparia* having the lowest response. In the present *V. riparia* study, *SnRK2.2* was up-regulated in both WD root and WD shoot, indicating a difference between isolated leaf dehydration and whole plant water deficit signaling.

### Cross-talk between ethylene, cytokinin, circadian rhythm and ABA signaling pathways

The expression profiles of the ABA, ethylene, cytokinin, and circadian rhythm signaling in response to water deficit in the shoot and root suggest WD induced hormone cross-talk [14, 86]. Coexpression networks constructed for leaf dehydration responses indicate key regulatory roles for ABA and ethylene signaling [14]. Similarly, networks constructed for hormone signaling in *V. riparia* WD roots and WD shoots had strong correlations of ABA with ethylene signaling with both *ERF* and AP2 domain-containing transcription factor/ERF up regulated in response to water deficit. In contrast, Auxin signaling did not have a strong correlation in *V. riparia* with ABA signaling, but auxin and ethylene signaling were more strongly related in both root and shoot under WD. The strong correlation of the ABA with the ethylene network found in WD root and WD shoot in the present study supports the role of cross-talk between ABA signaling and ethylene signaling and its proposed mediation by *ABF2* and *ABI5* identified in leaf dehydration studies [14]. It is of particular interest that although *ABF2* was strongly correlated in leaves and roots in the present study, *ABF3* was only correlated in the root, suggesting a root specific role for *ABF3*.

The potential for cross-talk between ABA and cytokinin is strong, as type-a arabidopsis response regulators (*ARR4, ARR5 ARR6*) interact with and regulate expression levels of the ABA signaling gene, abscisic acid insensitive 5 (*ABI5),* in addition to regulating cytokinin signaling [87]*.* Furthermore, three cytokinin receptor histidine kinases (*AHK2, AHK3,* and *AHK4*) are negative regulators of ABA signaling [88]. A bidirectional cross-talk is noted for ABA and circadian rhythm signaling [89, 90]. In *V. riparia* WD roots*, ABI5–1* is up-regulated and there are no *ARR* DEGs (negative regulator). In the WD shoot, *ARR4 and ARR5* (VIT_01s0026g00940 and VIT_17s000g07580) are significantly down regulated and *ABI5* is up-regulated. Hopper et al. [14] identified *ABF2* and *ABI5–1* as key ABA signaling hubs whose expression correlated with the drought tolerance of the leaves of three grapevine species. The up-regulation of *ABF2* and *ABI5*–1 in WD root and WD shoot in the current study support the proposed major role of these signaling genes in water deficit signaling in grapevine [14].

Circadian clock gating regulates ABA signaling networks [90]. *MYB96* transcription factor contributes to the gating of abscisic acid responses by binding to timing of cab expression 1 (*TOC1*) [76]. *TOC1* binds to the promoter of ABA related gene (*ABAR*) and *TOC1* is induced by ABA [91, 92]. Constitutive expression of clock-associated pseudo-response regulator (*PRR*) genes improves drought tolerance in *Arabidopsis* [93], supporting the hypothesis of the involvement of cytokinin signaling in response to water deficit. The common expression profile characteristics of the ABA, cytokinin, and circadian rhythm signaling in response to WD in this study, indicated that cross-talk between the hormone signaling and clock associated genes may play a role in water deficit response. The circadian clock has been strongly implicated in growth and development synchronization and changes that impact development of organs could be expected to show circadian perturbations. In particular, it has been noted that the circadian clock rephrases during lateral root initiation indicating a role of the circadian clock in adapting growth cycling to environmental conditions [94].

In this study, the circadian rhythm and ABA and cytokinin signaling showed strong correlations with *COL3* and *TOC1* expression in the WD root and WD shoot, respectively. In addition, *GI*, which is increasingly implicated in abiotic stress tolerance [86], was identified as a contributing member of both the WD root and shoot ABA, cytokinin and circadian rhythm cross-talk networks. While ABA and circadian rhythm gene interactions have been noted in ABA gating in the leaves in response to water deficit [90] or root growth in relation to carbohydrate availability [95], interactions of ABA, cytokinins, and circadian rhythms have not been previously reported in root in response to water deficits in grapevine. These results suggest a strong role for circadian clock mechanisms in grapevine root acclimation to soil water changes.

### Water deficit-responsive transcription factor profiles are different in WD root than WD shoot

Transcription factors greatly influence the plant’s responses to water deficits. The responses vary with genotype and organ, indicating different signaling networks are involved in the complex plant responses to water deficit. ABA-independent signaling pathways like *NAC* transcription factors are known to be involved in drought stress responses and modulate downstream early response to dehydration1 gene transcription [96, 97]. The NAC transcription factors are differentially expressed in grapevine in response to abiotic stress [98]. *NAC* transcription factors expression change in leaves in response to water deficit [26]*.* A large number of *NAC*s are differentially expressed in two rootstocks (M4 and 101.14) with 14 differentially expressed in both roots and leaves during water deficit treatments [18]. There were fewer differentially expressed in *V. riparia* in this study; however, eight of the genes that were DEGs in WD shoot and WD root are found in the Corso et al. [18] rootstock study in response to WD. Although there were more differentially expressed *NAC* transcription factor genes in the *V. riparia* WD shoot, in contrast, to the *NAC*s in M4 and 101.14 rootstock leaves. This suggests that while up-regulation of *NAC* transcription factors has been associated with increased drought tolerance, careful review of each *NAC* must be conducted by organ to identify this transcription factor’s role in drought tolerance. A *V. amurensis NAC029* (VIT_01s0026g02710) improves drought tolerance, modulates jasmonic acid synthesis, and enhances ROS scavenging enzymes in transgenic *Arabidopsis* [99]. In the present study, seven of the *V. riparia NAC* were up-regulated in both root and shoot in response to WD, suggesting a specific role for these particular *NAC* transcription factors in water deficit tolerance that may be related to ROS scavenging enzyme regulation.

*MYB*, a large transcription factor family, triggers the expression of drought- and ABA- induced genes to improve drought tolerance [100–102]. Ectopic expression of *OsMYB4* transcription factor is associated with physiological and biochemical adaptation to drought stress in apples [103]. In *V. riparia,* flavonoid biosynthesis was generally up-regulated in WD shoot in contrast to WD root. There were more differentially expressed *MYB* transcription factors in WD shoot than WD root in *V. riparia.* This was in contrast with M4 and 101.14 root, which had more *MYB*s differentially expressed in the roots than in leaves under water deficit [18]. There were more *MYB*s differentially expressed in *V. riparia* than M4 and 101.14; however, the *MYB*s that were common in the WD treated shoots of each of the three genotypes were up-regulated, suggesting a regulatory role for *MYB* in water deficit shoot.

## Conclusions

Mature leaf abscission, morphological root changes, and shoot growth cessation were found during the water deficit treatments and indicated that *V. riparia* may be reallocating its limited resources to maintain the apical portion of the grapevine shoot. An overall decrease in photosynthesis and an increase in dry matter content of roots demonstrated that *V. riparia* reallocated resources to sustain potential changes in the root that contributed to water maintenance in the early stages of water deficit. RNAseq transcriptome profiling of *V. riparia* revealed significant impacts of WD in roots and shoots. The larger number of DEGs and enriched VitisNet pathways found in WD shoot indicated that the shoot had a greater response to water deficit than the grapevine root. There were a large number of DEGs in response to WD. In addition, 10 VitisNet pathways were enriched in common with both WD root and WD shoot: notably, ABA biosynthesis, cytokinin signaling, *NAC* transcription factors and galactose metabolism. The transcript abundance of genes for enzymes involved in carotenoid and ABA biosynthesis (*ABA1*, *VDE,* and *NCED3)* increased in both WD root and WD shoot relative to their respective well-watered controls. There were more ROS DEGs in WD shoot than WD root, indicative of greater potential ROS scavenging action, most likely related to the photosynthetic capacity of the shoot. Impact on water movement capacity, as evidenced by aquaporin gene expression, presented two aquaporins that were up-regulated in the roots. In contrast, all aquaporin DEGs in the shoots were down-regulated. This suggested that aquaporins contributed to water uptake in the root and limited water loss in the shoot. Specific members of the *NAC* transcription factor family had similar responses and identified a common signaling role in both WD root and WD shoot. Key genes common in WD root and WD shoot were identified from the interacting hormone expression networks between ABA, ethylene, cytokinin, and circadian rhythm genes. These expression patterns suggested potential genes, as well as organ specific genes, to be explored in future regulatory time course studies. The combined shoot and root responses indicated the involvement of complex signaling networks in water deficit responses in grapevine roots as have been previously described for WD shoots [14]. In addition, the root expression data supported an ABA, cytokinin, and circadian rhythm signaling network in grapevine roots in response to water deficit.

## Additional files

Additional file 1: **Table S1.** RT-PCR candidate gene information. Candidate gene name, Unique ID, forward (F) and reverse (R) primer sequences, optimal primer concentrations (nM), amplification efficiency (%), and correlation coefficient for fold change of RT-PCR and RNAseq expression values (R2) of each gene in root and shoot. (XLSX 10 kb)

Additional file 2: **Table S2.** Root and shoot water and dry matter content. Mean % Water content (WC), hydration value (HV) and dry matter content (DMC) of grapevine tissues. Two-way ANOVA significance noted by: y = main effect of Day of Treatment; z = main effect of Water Status Treatment; yz = significant main effects of water status and day at *p* ≤ 0.05. (XLSX 9 kb)

Additional file 3: **Table S3.** Differentially expressed genes in water defict root (WD root) and well-watered root (C root). Values are normalized RPKM for each treatment, fold change (FC) and test statistic and q-value. Genes highlighted in gray were up-regulated in WD root relative to C root. Genes in bold were differentially express in common in WD root and WD shoot relative to their respective C tissue. (XLSX 176 kb)

Additional file 4: **Table S4.** Differentially expressed genes in water defict shoot (WD shoot) and well-watered shoot (C shoot). Values are normalized RPKM for each treatment, fold change (FC) and test statistic and q-value. Genes highlighted in gray were up-regulated in WD shoot relative to C shoot. Genes in bold were differentially express in common in WD root and WD shoot relative to their respective C tissue. (XLSX 673 kb)

Additional file 5: **Figure S1.** Shoot and root DEG in common during water deficit. Values are fold change (FC, log2) of water deficit root and shoot relative to their respective control. (PPTX 61 kb)

Additional file 6: **Figure S2.** Expression fold change for ABA metabolic and signaling genes in water deficit (WD) roots and shoot tips. All values were measured using real-time PCR and ratio of water deficit /control (WD/C)). Gene ids are *ABA1* (VIT_07s0031g00620), *NCED3* (VIT_19s0093g00550), *PP2CA* (VIT_13s0019g02200), *CYP707A3* (VIT_02s0087g00710), *ABA3* (VIT_19s0027g01090), and *ABI1* (VIT_11s0016g03180). Root = solid bar, Shoot = striped bars; values with asterix denote genes differentially expressed between tissues, with a *p*-value < 0.05 noted above bar (*n* = 3). (PPTX 43 kb)

Additional file 7: **Table S5.** Hormone signaling networks. A. ABA, auxin, and ethylene network for *V. riparia* root. B. ABA, auxin, and ethylene network for *V. riparia* shoot. 5C. ABA, cytokinin, and circadian rhythm network for *V. riparia* root. D. ABA, cytokinin, and circadian rhythm network for *V. riparia* shoot. (XLSX 16 kb)

Additional file 8: **Table S6.** RPKM expression fold change (FC log2) of reactive oxygen species scavenging enzymes in water deficit (WD) relative to well-watered organ (C) for root and shoot. Enzymes are noted as superoxide dismutase (SOD), glutathione reductase (GR), glutathione peroxidase (GPX), catalase (CAT), and ascorbate peroxidase (APX). Differentially expressed genes (DEG) at q-value < 0.05 are in bold lettering and highlighted according to organ in which DEG occured; root (gray), shoot (green), and both WD organs relative to their respective C (yellow). (XLSX 10 kb)

## Abbreviations

ABA: Abscisic acid
C: Control
DEGs: Differentially expressed genes
DW: Dry weight
FDR: False discovery rate
FW: Fresh weight
Pn: Net photosynthesis
SC: Stomatal conductance
WD: Water deficit
Ψ_stem_: Stem water potential
