# Supplementary figures and images for: Transcriptomic response is more sensitive to water deficit in shoots than roots of Vitis riparia (Michx.)
Source: BMC Plant Biol. 2019 Feb 13;19:72. doi: 10.1186/s12870-019-1664-7 (PMC6375209; doi:10.1186/s12870-019-1664-7)

## Slide 1
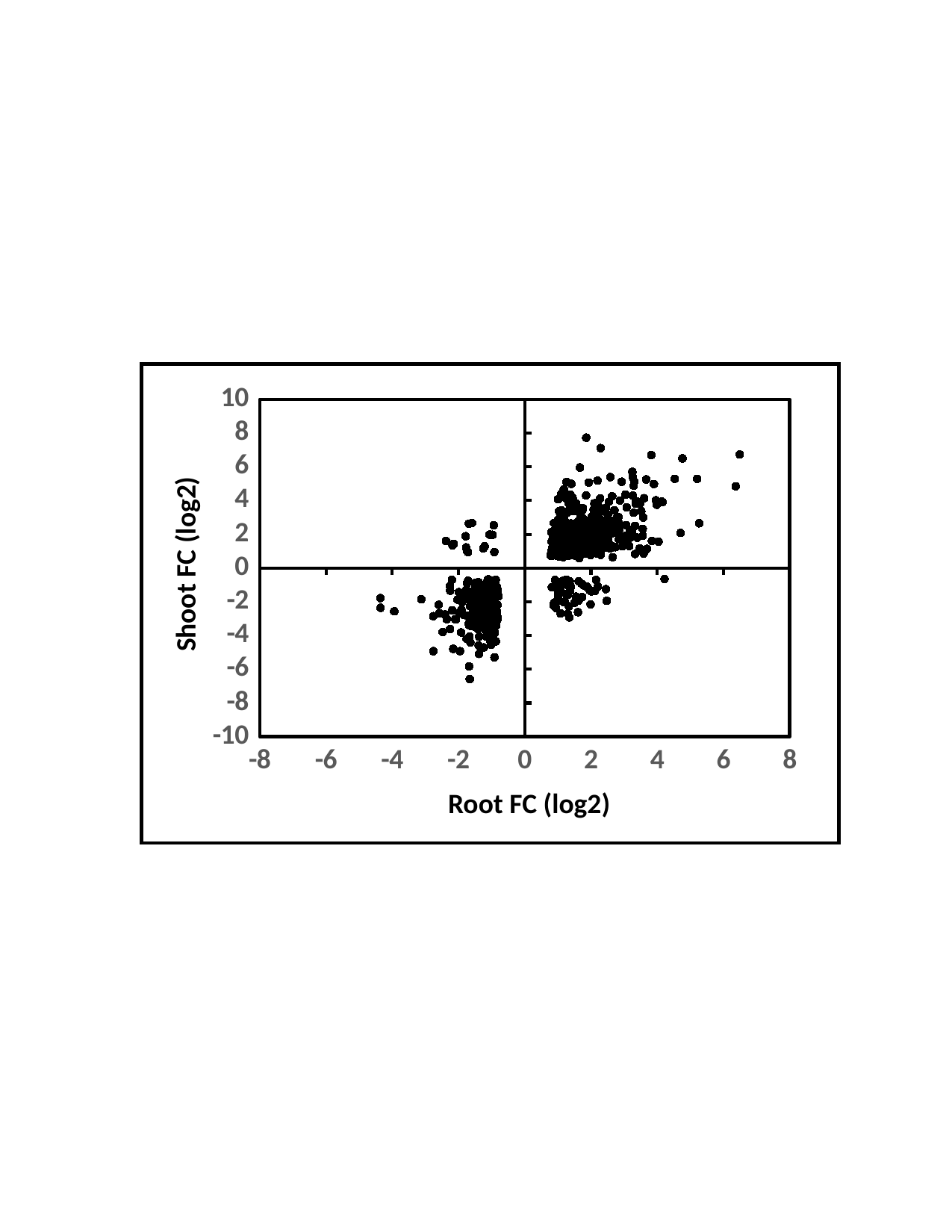

### Chart
| Category | shoot |
|---|---|Shoot FC (log2)
Root FC (log2)

Supplement: Supplementary file 5 — Figure S1. Shoot and root DEG in common during water deficit. Values are fold change (FC, log2) of water deficit root and shoot relative to their respective control. (PPTX 61 kb) [file 12870_2019_1664_MOESM5_ESM.pptx]
