# Supplementary material for: Transcriptomic response is more sensitive to water deficit in shoots than roots of Vitis riparia (Michx.)
Source: BMC Plant Biol. 2019 Feb 13;19:72. doi: 10.1186/s12870-019-1664-7 (PMC6375209; doi:10.1186/s12870-019-1664-7)
Supplement: Supplementary file 6 — Figure S2. Expression fold change for ABA metabolic and signaling genes in water deficit (WD) roots and shoot tips. All values were measured using real-time PCR and ratio of water deficit /control (WD/C)). Gene ids are ABA1 (VIT_07s0031g00620), NCED3 (VIT_19s0093g00550), PP2CA (VIT_13s0019g02200), CYP707A3 (VIT_02s0087g00710), ABA3 (VIT_19s0027g01090), and ABI1 (VIT_11s0016g03180). Root = solid bar, Shoot = striped bars; values with asterix denote genes differentially expressed between tissues, with a p-value < 0.05 noted above bar (n = 3). (PPTX 43 kb) [file 12870_2019_1664_MOESM6_ESM.pptx]

## Slide 1
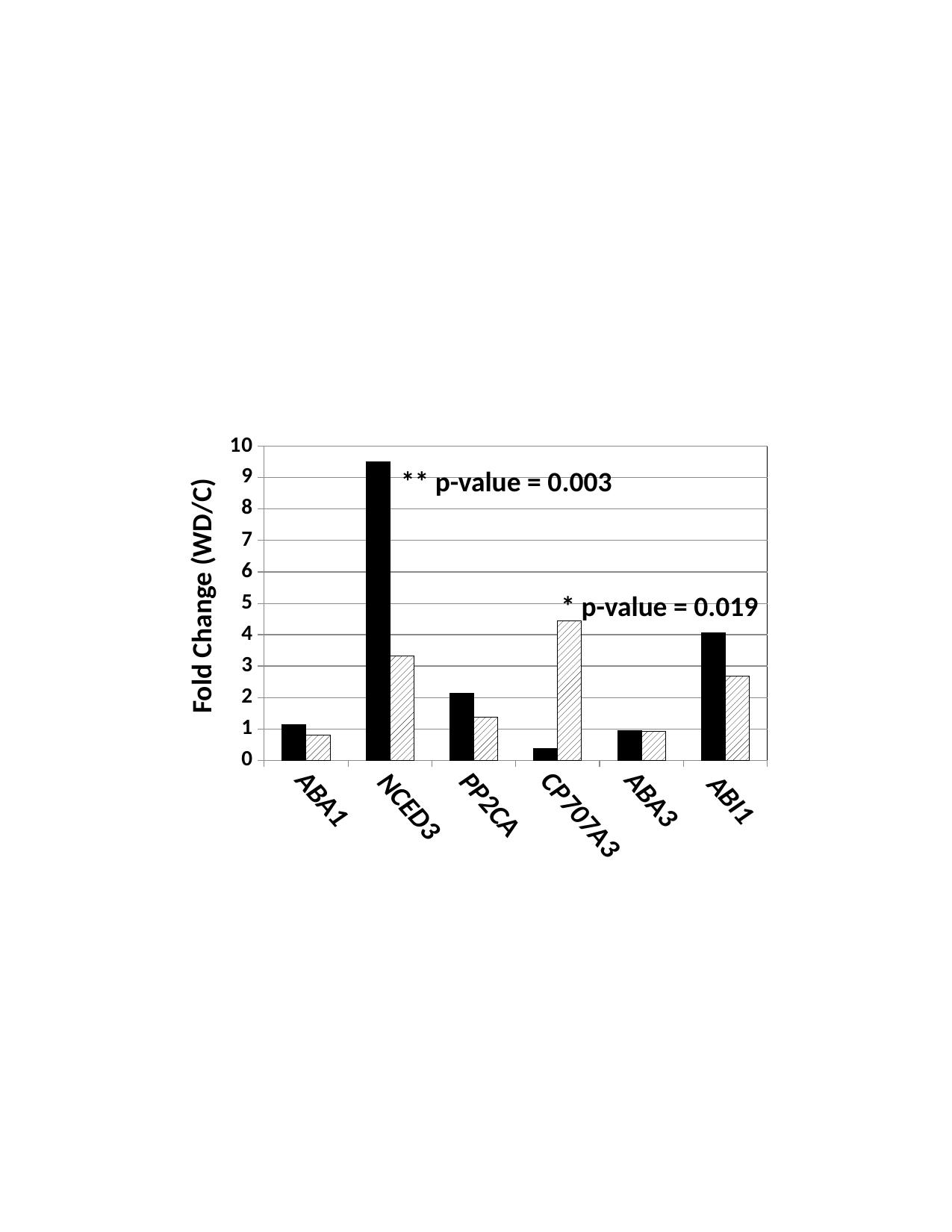

### Chart
| Category | Root | Shoot tip |
|---|---|---|
| ABA1 | 1.14 | 0.793 |
| NCED3 | 9.52 | 3.32 |
| PP2CA | 2.12 | 1.38 |
| CYP707A3 | 0.38 | 4.44 |
| ABA3 | 0.94 | 0.91 |
| ABI1 | 4.07 | 2.69 |** p-value = 0.003
* p-value = 0.019
Fold Change (WD/C)
ABA1
ABA3
ABI1
PP2CA
NCED3
CP707A3
